# Outbreak of *Listeria monocytogenes* in hospital linked to a fava bean product, Finland, 2015 to 2019

**DOI:** 10.2807/1560-7917.ES.2024.29.19.2300488

**Published:** 2024-05-09

**Authors:** Eveline Otte im Kampe, Saara Salmenlinna, Riikka Åberg, Suvi Wallgren, Maria Hautaniemi, Satu Keronen, Elina Leinonen, Annika Pihlajasaari, Eeva Ruotsalainen, Anna Sarvela, Ruska Rimhanen-Finne

**Affiliations:** 1 ECDC Fellowship Programme, Field Epidemiology path (EPIET), European Centre for Disease Prevention and Control (ECDC), Stockholm, Sweden; 2 Finnish Institute for Health and Welfare (THL), Helsinki, Finland; 3 Food Safety Unit, Environmental Services, City of Helsinki, Finland; 4 Finnish Food Authority (FFA), Helsinki, Finland; 5 Abdominal Center, Department of Nephrology, University of Helsinki and Helsinki University Hospital, Helsinki, Finland; 6 Division of Infectious Diseases, Inflammation Center, HUS Helsinki University Hospital, Helsinki, Finland; 7 Environmental office, City of Seinäjoki, Seinäjoki, Finland

**Keywords:** Listeria, food, outbreak, nosocomial, hospital, Finland

## Abstract

*Listeria monocytogenes (Lm)* is a bacterium widely distributed in the environment. Listeriosis is a severe disease associated with high hospitalisation and mortality rates. In April 2019, listeriosis was diagnosed in two hospital patients in Finland. We conducted a descriptive study to identify the source of the infection and defined a case as a person with a laboratory-confirmed *Lm* serogroup IIa sequence type (ST) 37. Six cases with *Lm* ST 37 were notified to the Finnish Infectious Diseases Registry between 2015 and 2019. Patient interviews and hospital menus were used to target traceback investigation of the implicated foods. In 2021 and 2022, similar *Lm* ST 37 was detected from samples of a ready-to-eat plant-based food product including fava beans. Inspections by the manufacturer and the local food control authority indicated that the food products were contaminated with *Lm* after pasteurisation. Our investigation highlights the importance that companies producing plant-based food are subject to similar controls as those producing food of animal origin. Hospital menus can be a useful source of information that is not dependent on patient recall.

**Key public health message**

**What did you want to address in this study and why?**

Listeriosis is a severe food-borne disease caused by infection with the bacterium *Listeria monocytogenes*. The disease can manifest as invasive illness, mostly in elderly people, pregnant women, unborn or newborn babies and people with weakened immune systems. Our aim was to identify the source of a hospital outbreak of listeriosis, initiate control measures and prevent further cases.

**What have we learnt from this study?**

The source of the outbreak was a ready-to-eat plant-based food product including fava beans, which to our knowledge has not been reported before. Listeriosis cases were linked to the product based on a backward-looking investigation that combined data on the hospital menu served to the patients during the incubation period and comparison of the *Listeria monocytogenes* isolates detected at the production plant and from the patients.

**What are the implications of your findings for public health?**

The risks involved in the production of plant-based products are the same as for products of animal origin. It is important that establishments producing plant-based food are subject to similar controls as those producing animal-based food. Foods more likely to be contaminated with *Listeria monocytogenes* should not be served to hospital patients.

## Background

Listeriosis is a severe bacterial infection caused by *Listeria monocytogenes* (*Lm*), usually via consumption of food contaminated with the bacterium. However, other ways of transmission than food-borne have been reported [1]. *Listeria* species are ubiquitous in the environment and many animals shed *Lm* in their faeces. Infections are often associated with raw, chilled or ready-to-eat (RTE) foods. Symptoms develop within 1–70 days after eating food contaminated with *Lm* [2]. The disease can manifest as severe, invasive illness, more likely in elderly people, pregnant women, unborn or newborn babies and people with weakened immune systems [3]. In these persons, listeriosis may present as septicaemia or meningitis and lead to high hospitalisation and mortality rates.

In 2021, an incidence of 0.5 per 100,000 population was reported for listeriosis in the European Union/European Economic Area (EU/EEA), while the incidence in Finland was 1.3 per 100,000 [4].

Listeriosis cases have been reported in healthcare facilities [5-11]. Two healthcare-associated *Lm* outbreaks have been reported in Finland during the last three decades. In 1999, a large hospital outbreak occurred with six deaths in 25 cases of invasive listeriosis. The source of the outbreak was butter which may have been contaminated after pasteurisation [12]. In 2012, RTE meat jelly was suspected to be the source of a hospital outbreak affecting 25 people [9].

## Outbreak detection

In May 2019, the Finnish Institute for Health and Welfare (THL) identified two cases of listeriosis with isolates of sequence type (ST) 37 clustering in the core genome (cg) multilocus sequencing (MLST) (cgMLST) from the Helsinki and Uusimaa Hospital District. From the THL laboratory database, we searched cases with similar sequence type from 2015 onwards.

Here we report results from an investigation that was initiated to identify the source of the infection and prevent further cases.

## Methods

### Surveillance of invasive listeriosis in humans

National surveillance for invasive listeriosis has been carried out in Finland since 1995. Clinical microbiology laboratories and treating physicians notify listeriosis cases to the Finnish Infectious Disease Registry (FIDR). The notification includes information on the date of sampling, gender, age and the hospital district of the residence of the case.

The laboratories send *Lm* isolates obtained from tissue samples or from fetuses, stillborn infants, neonates or mothers to THL. Since 2015, *Lm* isolates have been characterised by whole genome sequencing (WGS) [13].

### Monitoring and control of *Listeria monocytogenes* in food products and food-processing environments

The competent authorities are responsible for official control of food businesses to verify compliance of the food business operators (FBOs) compliance with the requirements set out in Food Law [14]. During the control visits, the authorities may take samples for verification of the control run by the FBOs. The FBOs take samples from the food business according to their Hazard Analysis and Critical Control Points (HACCP) plan [15]. Routine samples and samples taken in outbreak investigations are sent to local official food laboratories in Finland for *Lm* analysis [16]. The local official food laboratories send *Lm* isolates from food and some of the *Lm* isolates from food-processing environments to Finnish Food Authority (FFA) for further characterisation. The isolates are characterised using the same methods and schemes as at THL [13].

### Case definition

We defined a case as a person with laboratory-confirmed *Lm* serogroup IIa ST 37, clustering within a difference of seven alleles based on cgMLST according to the national cgMLST pipeline and notified to FIDR from 2015 onwards.

### Data collection and epidemiological survey

We conducted a descriptive study to identify the source of infection.

Since 2016, local public health authorities have been instructed to interview all domestic cases of listeriosis using a standard online questionnaire developed by THL. Information on age, gender, symptom onset, hospitalisation, death, sampling date and specimen type, pregnancy, underlying medical conditions, use of immunosuppressants and antacids, other medical treatments, dietary history for 2 weeks before symptom onset, food handling in the home and food purchase history, travel history and knowledge of listeriosis are collected in the questionnaire.

Clinical details of the cases were obtained from the patient interviews and patient information system of the Helsinki and Uusimaa Hospital District. Data on inpatient meals were collected from the hospital department menus.

We collected these data and stored them in Microsoft Excel (version 16.0).

### Microbiological and environmental investigation

A local food control officer made a routine inspection to the hospital kitchen on 3 May 2019. On 16 July 2019, the local food control officer took samples from the hospital kitchen after cleaning and disinfection. Ten surface samples were collected using a 3M Enviro Swab (3M, Maplewood, the United States (US)) from a worktable, handles of three drawers, a shelf where a disposable container was stored, a cutting board, two knife handles, a door handle of a cold storage room and a trolley of the diet kitchen. The samples were sent to a local official food laboratory and an analysis was initiated on the sampling day. The samples were analysed by real-time PCR using SureTect *Listeria monocytogenes* PCR Assay (Thermo Fisher Scientific, Waltham, US) and *Lm* was confirmed by a standard method (EN ISO 11290–1:2017).

Detection of *Lm* from clinical samples was performed at clinical microbiology laboratories using conventional culture methods. Isolates of *Lm* were identified by matrix-assisted laser-desorption-ionization time of flight (MALDI-TOF) mass spectrometry. Identification of ST and serogroup and clustering by cgMLST were performed as previously described [13].

### International outbreak inquiry

On 24 May 2019, THL posted an event notification on the Epidemic Intelligence Information System (EPIS) of the European Centre for Disease Prevention and Control (ECDC) to inquire if other countries had observed cases with the outbreak strain.

### Traceback investigation

In 2019, FFA and local food control authorities conducted a trace-back investigation of several different foods, such as salad and sandwich ingredients and margarines from different producers, consumed by the cases, based on the information from interviews and hospital menus.

## Results

### Descriptive epidemiology

**Figure 1:**
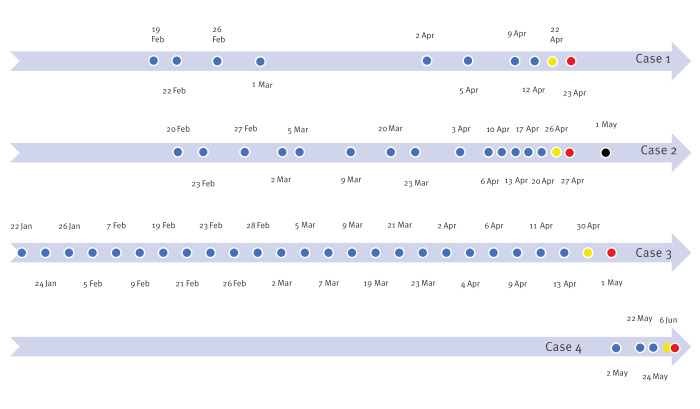
Confirmed cases of *Listeria monocytogenes* sequence type 37 in an outbreak linked to a fava bean product, by date of potential exposure, symptom onset and diagnosis, Finland, January–May 2019 (n = 4)^a^

In April–June 2019, four cases with *Lm* ST 37 (Case 1–4) were identified (Figure 1). In addition, one listeriosis case from 2015 (Case 5) and another from 2018 (Case 6) with isolates belonging to this cluster were identified, retrospectively. The ages of the cases ranged from late 40s to mid-70s. There were both men and women among the cases. Cases 1–4 were treated in two wards of a hospital in southern Finland, whereas the two cases in 2015 and 2018 were from eastern and western Finland, respectively.

Blue dot: potential exposure; yellow dot: symptom onset; red dot: diagnosis; black dot: death.

^a^ Information of cases (n = 2) from 2015 and 2018 is not included.

Cases had several underlying conditions such as kidney failure with need of maintenance dialysis, cancer, hypertension and diabetes mellitus type 2 or were otherwise immunocompromised. Two cases died within 15 days of their listeriosis diagnosis. One case had been hospitalised 14 days before symptom onset. Four had not travelled abroad within 14 days of the onset of illness, information on foreign travel was lacking from two cases.

Two cases could be interviewed using an online questionnaire, for two cases, clinical information was collected from the patient information system of the hospital district and data for two cases were not available. The interviewed cases reported consumption of frozen fruits, sliced ham and RTE salads within 2 weeks before the symptom onset.

During dialysis treatment, cases 1–4 were served snacks prepared by the hospital food service. The hospital menus of the days of treatment revealed the following common food exposures: cheese, chicken, tuna and a fava bean product salad, salad dressing, a sandwich with a fava bean product, margarine, egg spread, gherkins, tomatoes, ham and rice pasty. The cases could have been exposed to the suspected foods for 1–3 weeks before the onset of their symptoms (Figure 1). No information on potential food exposure was available for Cases 5 and 6.

### Microbiological investigation of environmental and food samples and comparison to patient isolates

**Figure 2:**
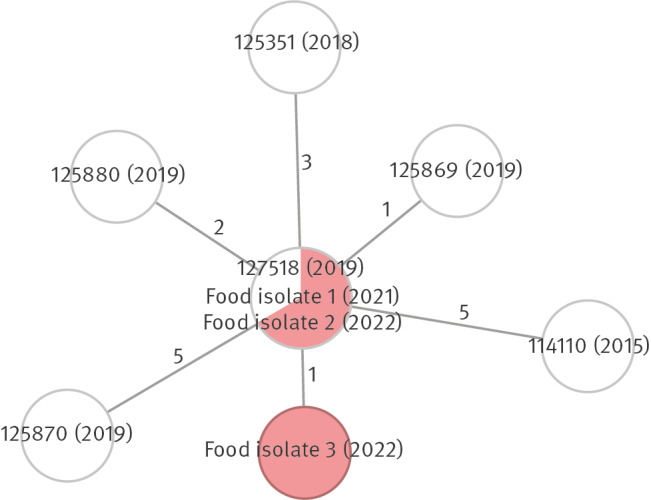
Minimum-spanning tree of *Listeria monocytogenes* sequence type 37 isolates from human cases and food samples in an outbreak linked to a fava bean product, Finland, 2015–2022 (n = 9)

No food served to the cases was available for sampling, neither was *Lm* detected from 10 surface samples taken from the hospital kitchen in July 2019. In November 2021, *Lm* was detected from a HACCP sample of a fava bean product (Food isolate 1) taken by the FBO, analysed at a local official food laboratory and whole genome sequenced at FFA. In August 2022, isolates from HACCP samples (Food isolates 2 and 3) of another fava bean product from the same company were sequenced at FFA. Food isolates 1–3 were similar to the isolates from Cases 1–6 (Figure 2). No *Lm* was isolated from fava bean product samples between 2019 and 2020. In December 2021, 591 surface samples were taken from the processing environment of the fava bean product company and *Lm* was detected from 41 samples, from floors and nearby surfaces and from a ripening machine.

Numbers of alleles are shown next to the branches (not proportional in length to allele differences). Red colour indicates an isolate from a fava bean product, white colour an isolate from a human case. The isolate identification number is shown in the bubbles.

### International outbreak inquiry

No matching isolates (< 8 cg allelic differences) were found in the *Lm* WGS data of The European Surveillance System (TESSy) (https://www.ecdc.europa.eu/en/publications-data/european-surveillance-system-tessy).

### Environmental and traceback investigation

During dialysis, the patients were served snacks not served to other hospital patients or staff. Snacks and meals for dialysis patients were prepared in the same kitchen preparing food for the hospital restaurant and other hospital staff. The same kitchen utensils, like cutting boards and refrigeration equipment were used for preparing food for dialysis and other patients. Except for snacks and margarine, no raw or processed foods used in the preparation of meals were served exclusively to dialysis patients. Any leftover food was discarded. Fresh meals were prepared daily and stored in sealed, disposable containers. Meals were transported to the dialysis patients in trolleys used by the kitchen and the trolleys were mechanically washed on return from the ward. Frozen foods used in the meals of dialysis patients were stored in a large freezer from which raw materials were retrieved as needed.

In the routine inspection of the hospital kitchen facilities on 3 May 2019, the local food control officer identified deficiencies in the cleanliness of storage, storage of cleaning equipment, equipment of the washbasin and the automatic temperature monitoring system for chilled and frozen food. The temperature of the refrigerator used for storing snacks and margarine for the dialysis patients was appropriate. In a traceback investigation conducted in 2019 no specific food was identified as the implicated food item.

The fava bean product was made from whole fava beans produced in Finland. The product was recommended to be consumed as such or for sauces, patties or baking. Based on the product label, the fava bean product was protein-rich, high in salt, packed in a modified atmosphere and stored refrigerated with a shelf-life of 24 days. No information on the water activity and pH value was available for the local food control authorities.

The large-scale production of the fava bean products and their availability to consumers had started in 2016. Previously, other food products were produced in the same facilities. Fava bean products produced by the company were sold throughout the country and were widely available to consumers.

Inspections by the manufacturer and the local food control officer indicated that *Lm* persisted in the production environment and entered the fava bean product after pasteurisation. The origin of the contamination was not found.

## Outbreak control measures

In 2019, the epidemiology unit of the Helsinki and Uusimaa hospital district informed the nephrology, haematology, rheumatology and cancer units about the listeriosis outbreak and listeria-risk food and ensured that a leaflet describing listeriosis and how to prevent infection was given to immunocompromised patients in these units.

During official controls at the production plant 2019–2021, deficiencies regarding labelling and HACCP sampling, cleanliness management of the facilities, separation of activities requiring different hygiene levels and the working hygiene of the employees were noted. In December 2021, measures to control *Lm* contamination in the food handling area included updated extensive HACCP sampling of *Lm* in the products and the production area, intensification of cleaning and disinfection procedures and further training of the staff. The local food control officer monitored the control measures.

In December 2021 and August 2022, the producer of the fava bean products recalled products from the affected batches and informed consumers of the *Lm* findings.

In November 2022, the producer was requested to add a heating requirement to the package labels for fava bean products, since the source of *Lm* in the products and in the production environment was not found. Finding *Lm* in the surface samples indicated that the cleaning and disinfection measures were not sufficient to control *Lm*. In March 2023, the heating requirement was extended to all products manufactured and packed in the facility, except for products that underwent sufficient *Lm* destruction treatment in their final packaging. The company demonstrated the absence of *Lm* per 25 g in the products tested at the end of the production.

## Discussion

In this paper, we have reported an outbreak of listeriosis in dialysis patients exposed to RTE plant-based products including fava beans. Our investigation suggests that the products, used in sandwiches and salad ingredients served during dialysis treatment days, were the likely source of listeriosis for Cases 1–4. Similar isolates of *Lm* were found in the case specimens and, retrospectively, in plant-based products including fava beans from the same manufacturer whose products had been used as an ingredient in the snacks served to the patients. This genotype has not been seen in any other food or human isolates. The snacks had been served several times over several weeks before the onset of the patients’ symptoms.

To our knowledge, plant-based products including fava beans have not been implicated as a source of listeriosis before. However, experimental studies have shown that pea-protein-based meat alternatives can support the growth of *Lm* [17]. Outbreaks of listeriosis in dialysis patients are also rarely reported. In Germany in 1994–1995, kidney transplant patients were infected with *Lm* most likely during dialysis, but the source of the outbreak was not identified [18].

Our findings have implications for the diet of dialysis patients, who are immunocompromised [19] and therefore at higher risk of invasive listeriosis [3]. Current guidelines in Finland for people with a weakened immune system recommend heating RTE foods before consumption or eating some RTE products well before their use-by date [20]. This outbreak emphasises the importance of this recommendation that also covers processed products of plant origin. We recommend including plant-based RTE products in national guidelines as a risk food for *Lm* and labelling appropriately so that consumers can follow the instructions. Previous studies have shown that *Lm* survives and proliferates on fresh vegetables [21,22], and RTE vegetables and sprouts have been increasingly associated with food-borne outbreaks, including listeriosis [23,24].

Environmental and traceback investigations showed that the contamination of the fava bean food product from the affected batches originated most likely from the food production environment of the processing plant. The production environment could have been contaminated by the raw materials used at the plant. As the product was pasteurised during production, it is unlikely that it contained *Lm* after pasteurisation. The technical equipment used after pasteurisation or during the automated packaging process could be a possible route of contamination of the products.

It is important that FBOs manufacturing RTE foods conduct *Lm* risk analyses and risk management and sample the processing equipment for *Lm* as part of their HACCP sampling scheme according to the guidelines on microbiological testing in the food industry [25]. The Finnish guidance on food and food production environment sampling for *Lm* recommends minimum sampling frequencies based on the nature and scale of operations [26]. According to the EU regulation on microbiological criteria [15], manufacturers must carry out studies to show that foodstuffs comply with the criteria for *Lm* in RTE foods that are able to support the growth of *Lm* throughout their shelf-life and monitor the processing environment and equipment for *Lm* as a part of their sampling plans. In a quantitative microbiological risk assessment model, 92% of invasive listeriosis cases for all age–gender groups were attributable to doses above 10^5^ CFU/g per serving [25]. However, a low concentration of *Lm* can cause listeriosis in highly susceptible individuals [27,28]. The approach in Finland is in line with the Codex Alimentarius Commission guidelines [29] and policy on *Lm* in RTE foods in Canada [30], while the US has established a zero-tolerance approach irrespective of public health risk [31].

A source of food-borne listeriosis outbreak has been detected via FBOs HACCP samples [32] [33] highlighting the importance of manufacturer’s HACCP sampling in outbreak investigations. In our study, *Lm* was detected in several surface samples from the production area of the fava bean products as part of the manufacturer's HACCP plan 2021–2022. However, in 2019, the control sampling at the production site was probably not sufficient to detect *Lm* contamination. In addition, no raw materials or surface samples were tested by the authorities at the production plant, as fava bean products were not suspected the source of the outbreak at that time. After *Lm* was found in the products, measures to control the contamination in the plant included extensive sampling of *Lm* in the products and the production area and intensification of cleaning and disinfection.

This study has some limitations. No data were available on how many dialysis patients were tested for *Lm* or how many dialysis patients were potentially exposed to the suspect food product between February and June 2019. However, between February and May 2019, approximately 44 dialysis patients per week were treated in the same dialysis unit as cases in this outbreak.

Information on food exposures was mainly collected from the menus of dialysis patients. However, no information was available if the patients consumed all the food items mentioned in the menus. We assumed that almost all the dialysis patients ate some of the snacks served. All cases in 2019 were served fava bean snacks several times before listeriosis diagnosis. The estimated incubation period of 1–3 weeks was in line with the incubation period in previous reports [2]. While fava bean products probably were the source of the infection in four cases, no information was available on possible exposure to the products for the cases in 2015 and 2018. The case in 2018 could have consumed fava bean products while the case in 2015 might have consumed other products from the same manufacturer [34].

Samples of food served to the dialysis patients were not available for sampling in the hospital kitchen because the food was discarded immediately. Environmental samples were collected during an inspection of the hospital kitchen in July 2019, 2 months after the outbreak was detected. However, the hospital central kitchen was subject to regular inspections by the local food control authority in accordance with the EU Food Law and national regulations [14,35]. In 2020, THL and FFA published advice for institutional kitchens to remind the food safety authorities and those responsible for institutional kitchens about listeriosis and food products considered at risk [36,37]. Since *Lm* can be controlled through cooking, FFA has developed general instructions on safe handling of food for the general population [20]. However, many hospitals worldwide do not have food preparation policies or practices to minimise the risk of *Lm* contamination [38]. Given the potentially serious consequences of listeriosis in hospitalised patients, guidelines for food served in hospitals and long-term care facilities should be evaluated.

The size of this outbreak is most likely underestimated due to undetected cases with no or mild symptoms from which no samples were collected for laboratory confirmation. It is possible that products with *Lm* have entered the market despite HACCP sampling at the production site. Despite several years of national distribution of the product, no other cases have been detected in Finland. A possible explanation could be that the novel fava bean product is more popular among younger age groups, who tend to be in better health and are less susceptible to serious illness [3].

## Conclusion

Our investigation shows that hospital menus can be a useful source of information that is not dependent on patient recall. The evidence associating the RTE fava bean products with listeriosis was based on a retrospective descriptive analysis linking the menu served to the dialysis patients during the incubation period, the *Lm* strains detected at the production plant, and the patient strains. Contamination of the RTE fava bean product after pasteurisation during processing or packaging is the likely cause of this listeriosis outbreak. To prevent future cases, measures have been taken at the production plant to control the listeria contamination. A root-cause analysis might be able to highlight necessary measures to prevent the contamination of RTE foods with *Lm* in the future. Our investigation highlights that the risks involved in the production of plant-based products are the same as for animal products, and it is important that establishments producing plant-based food are subject to similar controls as those producing food of animal origin.
